# The role of video-assisted feedback sessions in resident teaching: A pre-post intervention

**DOI:** 10.15694/mep.2019.000219.1

**Published:** 2019-11-29

**Authors:** Allen Tran, Jaclyn Vertes, Tasha Kulai, Lori Connors

**Affiliations:** 1 Division of Medicine; 2 Department of Internal Medicine; 3 Department of Medicine; 4 Departments of Medicine and Pediatrics

**Keywords:** Medical education, Video-assisted feedback, Resident teaching, Self-reflection, Competence by Design

## Abstract

This article was migrated. The article was marked as recommended.

**Purpose:** Despite providing a large component of teaching to trainees, internal medicine residents receive little feedback on their teaching ability.

**Methods:** This was a single-center, mixed methods study of 19 senior internal medicine residents in Canada. Classroom-based teaching sessions delivered by the participants were individually video recorded. The individual recording was then watched by the participant and by two feedback facilitators, who then met for face-to-face feedback. Participants completed a self-reflective exercise after this intervention. Audience members of the recorded session and a post-feedback teaching session completed an evaluation form. Scores from the evaluation forms from each phase were analyzed with the Wilcoxon Signed-Rank Test. Inductive coding was performed for qualitative data from the feedback sessions and reflective exercises.

**Results:** 19 residents participated. There was no statistical difference in the evaluation form scores between the pre-intervention and post-intervention teaching sessions. Mean scores varied from 4.6 to 5.0 out of 5.0 on combined pre-and post-intervention evaluations. 89% of participants found viewing their recorded session useful. 94% of residents stated the intervention was worth continuing. Common themes of feedback and self-evaluation included “time-management,” “organization,” “communication,” and “environment.”

**Conclusion:** Video-assisted feedback of teaching improved self-perception of a resident’s teaching ability.

## Introduction

Medical residents play an integral role in the health care education of medical trainees. However, training and feedback on providing medical education is lacking from many residency training programs. A survey of Canadian pediatric residents revealed that only 35% had received any form of training on medical education ( Walton and Patel, 2008). A separate survey of American emergency residents found a similar rate of only 30% ( Riddell *et al*., 2017).

Although the importance of feedback on trainee performance is well recognized, there continues to be a lack of quality feedback to internal medicine residents. Written feedback by attending doctors of internal medicine residents was primarily of low or moderate quality in a recent study ( Jackson *et al*., 2015). Notably, there were no comments made on resident teaching. Furthermore, the optimal method of delivery of feedback is unclear. A direct observational and feedback program by faculty of internal medicine residents’ teaching on bedside rounds resulted in 80% of residents reporting improvement in teaching by self-assessment ( Chandler *et al.*, 2012). The program also increased residents’ motivation to tailor their teaching to the feedback received.

Feedback supplemented with video recording of a trainee’s performance has shown educational value in various studies. For example, medical students have demonstrated significantly greater improvement in suturing performance upon reviewing their videotaped performance with feedback compared to standard lecture feedback (Craig I. Nesbitt *et al*., 2015). Video-assisted feedback has demonstrated benefits in procedural based skills in both undergraduate (Spence *et al*., 2016) and postgraduate trainees ( Soucisse *et al*., 2017). In addition, there is evidence that video-assisted feedback can also improve history and physical examination skills in medical students ( Paul *et al*., 1998; Nilsen and Baerheim, 2005). However, there is a paucity of literature available on the value of video-assisted feedback on improving a resident’s teaching skill.

Questions remain on the optimal content, delivery, and duration of teaching programs for residents. Programs geared towards providing formal training to residents (“Residents as Teachers”) appears to improve teaching effectiveness of residents, but these programs are heterogenous and it is unclear if there is a specific intervention that provides the largest impact ( Hill *et al*., 2009). Video recording of faculty is effective in reviewing teaching behaviours and allows for self-assessment and demonstrable improvement with time ( Pinsky and Wipf, 2000). We sought to address this gap in Canadian postgraduate medical education and address the values of Competence by Design (CBD), the Royal College of Physicians and Surgeons of Canada’ (RCPSC) recently initiated program that focuses on improving resident learning (The Royal College of Physicians and Surgeons of Canada :: Competence By Design). This study’s purpose was to determine if video recording of a resident’s teaching session, followed by formal feedback and reflective exercises, led to improvement in a resident’s teaching skills.

## Methods

### Design

This was a pre-post intervention design that was conducted over the 2016 - 2017 academic year at an academic teaching hospital in Canada. Research ethics board approval was obtained. All recruited participants provided written informed consent.

### Population

The study population consisted of 19 senior internal medicine residents (Postgraduate year [PGY] 2 or 3) who were on a Medical Teaching Unit (MTU) rotation during the study period. MTU is an internal medicine ward-based rotation with a hierarchical team structure. Participating residents were recruited at the internal medicine academic half day by the investigational team.

### Intervention

The intervention took place for each resident during their MTU rotation where five, 30-minute, classroom-based teaching sessions per week are provided by senior residents. The audience consists of rotating medical students (third- and fourth-year students), junior residents (PGY1), senior internal medicine residents (PGY2 and PGY3), and attending physicians. The content of the teaching session is aimed at the internal medicine PGY1 level.

First, the participant had a teaching session video recorded early in the rotation. Then, the participant watched the video recording, as did two feedback facilitators. Next, the participant met with the two facilitators for feedback regarding the participant’s teaching session. The structured feedback sessions were facilitator-led and based on Agenda-Led, Outcome Based Analysis (ALOBA) principles ( Silverman *et al*., 1997). The facilitators encouraged self-assessment first and then provided their own feedback to the participant. This session was audio recorded for qualitative data to identify themes of feedback. Afterwards, the participant completed a self-reflective exercise regarding their perception on the utility of watching the video recording and the feedback session ( Table 1).

Standardized evaluation forms were completed by audience members during the recorded teaching session and at a teaching session that occurred after the participant had their feedback session ( Table 2). These forms included both Likert scales and an area for free text. The PGY2s and PGY3s on the rotation did not fill out evaluation forms as they would be potential participants in the intervention. Attending staff also did not fill out evaluation forms as attendance at these sessions is quite variable by this group at our institution. The evaluations may not have come from the same group of audience members due to uncontrollable variables leading to audience member absence from the teaching session, such as post-call days and vacation.

### Feedback Facilitators

Facilitators were residents who completed a medical education resident elective. They were in their PGY4 or PGY5 year of training who were taught guidelines and evidence surrounding medical education and providing feedback. All facilitators met prior to the commencement of the study period to standardize the feedback framework. This was led by author LC, who has a master’s degree in medical education.

### Outcomes

The primary outcome was to determine if the multi-faceted intervention would improve the participant’s evaluation scores from the audience. The secondary outcome was subjective improvement in the participant’s self-evaluation of their teaching skills.

### Analysis

The evaluation forms provided quantitative data. The pre-intervention and post-intervention means were analyzed with a Wilcoxon Signed-Rank Test for each question on the evaluation form for each participant. Analysis was carried out in SPSS Statistics, version 24 (IBM Inc., Armonk, NY, 2016).

Qualitative data was obtained from the free text area of the evaluation forms, written self-reflective exercise, and audio recordings from the feedback sessions. Inductive coding and thematic analysis to saturation were done independently by authors AT and JV.

## Results/Analysis

A total of 22 residents provided informed consent to participate. Three did not participate due to logistical reasons (unavailability of study team members to provide feedback or video record teaching sessions) or because the resident did not have at least two scheduled teaching sessions during the MTU rotation. The final study population consisted of 19 residents, of which 8 were in their PGY3 year ( Table 1). One participant did not have a post-intervention evaluation completed due to availability of study team members to distribute and collect the evaluation forms. One other participant did not complete a self-reflective exercise. Thus, 18 participants were included in the quantitative analysis and 18 participants provided qualitative data. There was a mean of 11 evaluations per teaching session.

**Table 1.** Baseline characteristics of the residents

	Number (n = 19)
Male	12 (63%)
PGY3 status	8 (42%)
Past training on medical education in any form *	8 (42%)
Any past feedback on teaching *	10 (56%)

* N = 18 as 1 participant did not provide this data in the self-reflective exercise. PGY3 = Postgraduate year 3.

### Evaluation form

**Table 2.** Mean evaluation form scores among the residents prior to and after the intervention (n=18)

Question	Mean *	P-value
1) The resident spoke clearly		
Pre-intervention	4.8	0.17
Post-intervention	4.8	
2) An open learning environment was encouraged		
Pre-intervention	4.7	0.69
Post-intervention	4.7	
3) The presentation was well organized and was easily followed		
Pre-intervention	4.7	0.54
Post-intervention	4.8	
4) Teaching seemed to be at an appropriate level for the audience		
Pre-intervention	4.8	0.28
Post-intervention	4.8	
5) There was a good balance between participation and lecturing		
Pre-intervention	4.7	0.52
Post-intervention	4.6	
6) The resident appeared to know the content well		
Pre-intervention	4.8	0.84
Post-intervention	4.8	
7) I feel like I will be able to retain some learning points from this presentation		
Pre-intervention	4.7	0.51
Post-intervention	4.8	
8) Overall, the presentation was excellent:		
Pre-intervention	4.7	0.83
Post-intervention	4.8	

* Scores were on a 5-point Likert scale

Overall, there was no significant difference between pre-intervention and post-intervention evaluation scores ( Table 2). Common themes in the free text section of the evaluation form centered around “communication,” “presentation style,” and “content.” Many of these themes were evenly distributed between the pre-intervention and post-intervention periods.

“Communication” was a common theme in the free text section of the evaluation form. Speaking more clearly or slowly was a frequent comment. However, the participant having spoken clearly was almost as frequently mentioned. Difficulty reading the whiteboard, whether due to legibility or poor markers, occurred often.

Another common theme was “presentation style.” A comment on the participant being well organized emerged. The ability to encourage participation occurred more times than comments on the need to encourage more participation. There were more comments surrounding how well the resident encouraged participation in the pre-intervention phase. Conversely, the need for better audience participation was noted more frequently in the post-intervention phase. Preference of whiteboard teaching rather than slide-based teaching was noted with no mention of the converse. High quality images appeared to be noteworthy. The need for more summarization seemed to be as prevalent as a comment regarding excellent summarization by the participant.

Within the “content” theme, the evaluators frequently stated the topic was high yield. Many also found that the topics were relevant to their level of training. There was a strong desire for more practice cases being incorporated, as well as discussion around management of conditions. Another common criticism revolved around too much content being discussed in the allotted time, and this theme was stronger in the pre-intervention audience comments.

### Feedback session

One theme from the feedback session related to “presentation skills”. Communication, such as speaking too quickly and mumbling, was often discussed. In addition, participants realized that they were facing the whiteboard or their notes more often than facing the audience. Another theme among feedback was “setting up the environment”. This revolved around ensuring there was optimal lighting for a whiteboard presentation versus a slide-based presentation. It also included reorientation of the chairs in the room. One other feedback theme was “organization”. Many participants did not clearly set session objectives, which then led to issues with time management. Sign-posting and summarization were mentioned often as feedback to improve the structure of the sessions.

### Self-Reflections

Sixteen (89%) participants found watching the video recording of their teaching sessions useful. Ten (63%) reported that it was helpful to view from the audience’s perspective. “It let me see myself how the audience actually sees me and not how I assume they see me during teaching,” explained one participant. This facilitated awareness of distracting body language/mannerisms, as well as voice projection and audience focus. One participant noted this perspective made “feedback more tangible”. Watching the video recording helped three (19%) participants with viewing their usage of the whiteboard. One (6%) participant did not find the recording useful and found the feedback session to be more worthwhile. One (6%) participant somewhat found the recording useful as it improved confidence by allowing the participant “to see myself not doing a completely terrible job”.

When asked what the participant learned from watching their own video recording, there was an emergence of themes similar to the feedback session themes. These centered around “non-verbal communication,” “verbal communication,” “time-management/planning,” and “style”. These same themes were identified as changes the participants would make after experiencing the intervention. After viewing the video recording, one participant reflected, “I had a schemata in my head of how the teaching would go and I see now how I could have tailored it better.”

The feedback session was found to be useful by 17 (94%) of the residents. Frequent comments included receiving “useful feedback” (35%) or feedback that was not identified upon self-reflection (41%). One participant stated, “The feedback providers had suggestions on domains of teaching that I had not considered. Things like room set up and modifying body language to encourage participation.” There were two (11%) comments that feedback was too generalized. However, one of these participants noted that “the practice of going through the feedback process encouraged self-reflection”.

Seventeen (94%) participants reported that they thought this intervention was worth continuing. Four (24%) participants particularly valued the prompt feedback. One (6%) participant stated that this intervention should be coupled with a formal teaching course. Two (11%) participants were concerned about the sustainability of the time-intensive intervention. The concern of feedback being too generalized and potentially “homogenizing teaching styles” were mentioned by two (11%) participants. One (6%) participant stated, “It seems as though it is overall helpful for our own learning; there isn’t necessarily a scale to know if we take anything away from it, which would be helpful.”

## Discussion

Video-assisted feedback of resident teaching did not lead to improvements in audience evaluation scores. However, the videos allowed for self-reflection and were positively received by residents as a tool to improve teaching ability. This study sheds light on the benefits of video-assisted feedback on improving teaching skill by residents.

A difference in evaluation of teaching scores was not detected after the intervention. Average scores were high in both the pre-intervention and post-intervention evaluations, which likely contributed to the lack of difference. This could be for two reasons: one being that evaluators were medical students and junior residents, who may not be as critical compared with senior residents or attending physicians. Another potential reason may be related to evaluators being junior members on the medical team of the participants; there may have been a sense of pressure to provide high ratings due to the power differential. To ameliorate this, anonymity on the evaluation forms was maintained and participants were provided with a summary of the evaluations rather than individual forms.

This study elucidated common challenges experienced by the participants at this single center. Many participants struggled with time management, organization, and environment optimization. Simple suggestions, such as focusing on a clear, short set of objectives and summarization at the end of the session, were common. Also, it was common for participants to acknowledge that communication could be improved by speaking clearly or writing legibly. Many participants also did not previously consider environmental optimization, such as ensuring adequate lighting and audience chair orientation. Arguably, these deficiencies would not be self-identified without the participant watching their own video recording.

There were two comments from participants that feedback was too generalized, raising the possibility that this could result in “homogenizing teaching styles”. Indeed, themes were similar when analyzing data from the feedback sessions and from what the participants learned from the intervention. This could be due to many of the participants having common deficiencies, or it could be due to the feedback facilitators only noticing similar areas for improvement. This is an important criticism as medical teaching needs to be flexible to cater to the various learning styles of the audience. As well, certain topics may be best delivered by different styles of teaching.

This study demonstrates that internal medicine residents observing a video recording of their own teaching was positively perceived and that the majority found it beneficial. Observing a recording of oneself offers a unique method of detecting error, which is known to be a powerful feedback tool ( Hattie and Timperley, 2007). Studies involving medical students have also found that viewing their own video recorded history and physical examination was a welcome modality of feedback as it provided a new platform for self-reflection ( Paul *et al*., 1998; Nilsen and Baerheim, 2005). Hermann-Werner *et al*. (2006) found that medical students subjectively rated their ‘learning success’ higher after video feedback. Video recordings have also been shown to improve the quality and detail of feedback in surgical literature, as they allow for identification of specific areas of performance that can be better developed ( Eubanks *et al*., 1999).

There are numerous studies that have used video recordings in medical education. Many of them relate to improving a trainee’s procedural skills (Craig I Nesbitt *et al*., 2015; Spence *et al*., 2016; Soucisse *et al*., 2017), but few relate to improving teaching ability. Lawson and Harvill found a 13-hour teaching course over 13 weeks improved resident teaching based on standardized evaluation of video recorded teaching sessions ( Lawson and Harvill, 1980). Their intervention also included self-evaluation via video recording. However, the teaching sessions that were recorded appeared to be within the context of the teaching course. The recordings in our study were done in situ rather than in a teaching course amongst peers. Our study’s recordings likely provided a better representation of the resident’s true baseline teaching abilities. This study contributes to the paucity of data in developing a resident’s teaching ability.

The feedback session was generally well-received by the participants. Interestingly, there were no negative comments regarding the anticipation of feedback, as this tension between wanting and fearing feedback has been described amongst learners ( Mann *et al.*, 2011). An external source providing an evaluation of one’s skills was valued and appeared to add a perceived incremental benefit. This is not surprising as there is difficulty in obtaining accurate self-assessment ( Dunning *et al*., 2004; Davis *et al.*, 2006). Self-assessment alone has been shown to have a limited effect on change in clinical teaching (Dunning *et al.,* 2004). Timely feedback, however, can be beneficial. When feedback is provided on a well-defined, repeated task, such as the resident-led teaching sessions in this study, it allows for more deliberate practice and improved performance ( Ericsson *et al*., 1993).

By incorporating a prompt feedback session and self-reflective exercise after the video recording, this study aimed to facilitate more extensive self-assessment. Feedback to clinical teachers is useful when it stimulates reflection ( Boerboom *et al.*, 2015). Feedback best enables self-assessment when it is timely and specific and provided by credible facilitators in a private environment (Bing-You *et al.,* 1997). As Epstein *et al*. (2008) note: “The power of self-assessment lies in two major domains - the integration of high quality external and internal data to assess current performance and promote future learning, and the capacity for ongoing self-monitoring during everyday clinical practice.” By providing various modalities of external data through video-recordings and feedback sessions, participants were able to engage in more meaningful self-assessment in this study, which is an important skill for clinicians to acquire that necessitates practice.

There were several limitations to this study. First, this was a resource-intensive intervention, which limits the ability to easily implement this strategy. Second, it was a multi-faceted intervention and it is not clear which aspect(s) contributed the most or were not beneficial. Third, while the self-reflective exercises were anonymized, this study was conducted by senior resident colleagues and a staff physician of the participants, which may have positively biased the participants’ responses to the usefulness of this intervention. Fourth, there is no “gold standard” for measuring teaching ability, which makes it difficult to compare the magnitude of effect from this study to existing literature. Fifth, we did not video record a teaching session after the intervention to determine if either the participant or the feedback facilitators noted any improvement or incorporation of feedback. This was due to time-constraints from this already resource-intensive intervention. Additionally, the participants did not present on the same topic again, which makes some aspects of comparison more difficult.

## Conclusion

Although our study showed no significant improvement in evaluation scores of resident teaching after the intervention, it did show a strong positive perception of video-assisted feedback in improving resident teaching. The majority of residents gained new insight into their teaching skills through viewing the video recording. This modality provided a new platform for self-reflective practice. Overall, video-assisted feedback sessions may have an important role in improving resident teaching, which in turn could enhance medical education for junior learners as a downstream effect, thereby highlighting the key values of CBD.

## Take Home Messages

• Internal medicine residents observing a video recording of their own teaching was positively perceived; the majority found it beneficial.
• Video-assisted feedback of teaching improved self-perception of a resident’s teaching ability.
• Video-assisted feedback sessions may have an important role in improving resident teaching, which in turn may enhance medical education for junior learners.

## Notes On Contributors

Dr. Allen Tran is a general internist with interests in thrombosis, point-of-care ultrasound, and medical education. He is the program director for the general internal medicine subspecialty training program at Dalhousie University.

Dr. Jaclyn Vertes is a fourth year internal medicine resident at Dalhousie University. She studied medicine at Trinity College, Dublin.

Dr. Tasha Kulai completed her internal medicine and gastroenterology residencies at Dalhousie University. She went on to complete a fellowship at the Mayo Clinic in Rochester, USA in Gastroenterology and Hepatology.

Dr. Lori Connors is a clinical immunologist and allergist with both academic and community-based practices. She is actively involved in curriculum development and teaching of clinical skills, and is assistant director of clerkship for Internal Medicine and program director for Pediatric Clinical Immunology and Allergy.
