# Supplementary material for: The role of video-assisted feedback sessions in resident teaching: A pre-post intervention
Source: MedEdPublish (2016). 2019 Nov 29;8:219. [Version 1] doi: 10.15694/mep.2019.000219.1 (PMC10712475; doi:10.15694/mep.2019.000219.1)
Supplement: Peer review history

# Reviewer report: Reviewer response for version 1

**DOI:** 10.21956/mep.19923.r30202

**Published:** 2020-01-17

**Authors:** Mary Dankbaar

**Affiliations:** 1 Erasmus University Medical Centre

This review has been migrated. The reviewer awarded 4 stars out of 5 Providing the opportunity for video-based self-reflection to improve teaching ability is an interesting intervention. It is also resource-intensive, as the authors underline. this study did not demonstrate an improvement in pre/ post evaluation scores on teaching ability; this may be due to a ceiling effect in the pre evaluation scores, due to non-anonymous responses from medical students and junior residents for the more senior teachers. Anonymous feedback and a larger sample size would be an important improvement for a next study.

Reviewer Expertise:

NA

No decision status is available

# Reviewer report: Reviewer response for version 1

**DOI:** 10.21956/mep.19923.r30206

**Published:** 2019-12-04

**Authors:** Felix Silwimba

**Affiliations:** 1 University of Lusaka

This review has been migrated. The reviewer awarded 5 stars out of 5 this paper is informative and educative to medical educators and use of technology in training medical educators. its excellent that it discuss the training of residents in medical education. I recommend it to all

Reviewer Expertise:

NA

No decision status is available

# Reviewer report: Reviewer response for version 1

**DOI:** 10.21956/mep.19923.r30205

**Published:** 2019-12-04

**Authors:** Trudie Roberts

**Affiliations:** 1 Leeds Institute of Medical Education

This review has been migrated. The reviewer awarded 4 stars out of 5 I think this paper is interesting in that it shows that the participants themselves found the exercise itself useful to them and the nurturing of a community of teachers is a very positive thing. It would be useful to video staff at the being and end of their rotations to see if they, themselves, perceive any changes in their teaching

Reviewer Expertise:

NA

No decision status is available

# Reviewer report: Reviewer response for version 1

**DOI:** 10.21956/mep.19923.r30204

**Published:** 2019-11-29

**Authors:** Balakrishnan Nair

**Affiliations:** 1 Centre for Medical Professional Development and University of Newcastle

This review has been migrated. The reviewer awarded 4 stars out of 5 We learn by reflection and anything to facilitate this process will help us . I found this study interesting from this point. The participants were all in the medical education unit and may have been highly motivated to get a high pre - intervention score. When we are being “watched “ there may be a Hawthorn effect too. A discussion about these will strengthen the paper

Reviewer Expertise:

NA

No decision status is available

# Reviewer report: Reviewer response for version 1

**DOI:** 10.21956/mep.19923.r30203

**Published:** 2019-11-29

**Authors:** Ian Wilson

**Affiliations:** 1 International Medical University

This review has been migrated. The reviewer awarded 4 stars out of 5 I found this an interesting paper as it demonstrated the acceptability of recording educational ssession provided by PGY 2 & 3 in internal medicine. Despite its acceptability it did not show any positive benefit in audience scores. The authors discuss some of the reson why this may be. To me thereseems to be s significant floor effect with the subjects all receiving high scores in the pre-intervention assessment. this leaves very little room in which to demonstrate a positive change.The amin strength of this paper is the demonstration of the the acceptability of self reflection on watching onself perform. this is itself is a significant finding for this institution.

Reviewer Expertise:

NA

No decision status is available
